# Gut resistome profiling reveals high diversity and fluctuations in pancreatic cancer cohorts

**DOI:** 10.3389/fcimb.2024.1354234

**Published:** 2024-02-07

**Authors:** Xudong Liu, Kexin Li, Yun Yang, Dingyan Cao, Xinjie Xu, Zilong He, Wenming Wu

**Affiliations:** ^1^The State Key Laboratory for Complex, Severe, and Rare Diseases, Peking Union Medical College Hospital, Beijing, China; ^2^School of Engineering Medicine, Beijing Advanced Innovation Center for Big Data-Based Precision Medicine, Interdisciplinary Innovation Institute of Medicine and Engineering, Beihang University, Beijing, China; ^3^Microbiome Dynamics, Leibniz Institute for Natural Product Research and Infection Biology- Hans Knöll Institute, Jena, Germany; ^4^Department of General Surgery, Peking Union Medical College Hospital, Chinese Academy of Medical Science & Peking Union Medical College, Beijing, China

**Keywords:** pancreatic cancer, gut microbiomes, antibiotic resistance genes, vancomycin-resistant genes, public pancreatic cancer cohorts

## Abstract

**Background:**

Pancreatic cancer is one of the deadliest cancer, with a 5-year overall survival rate of 11%. Unfortunately, most patients are diagnosed with advanced stage by the time they present with symptoms. In the past decade, microbiome studies have explored the association of pancreatic cancer with the human oral and gut microbiomes. However, the gut microbial antibiotic resistance genes profiling of pancreatic cancer patients was never reported compared to that of the healthy cohort.

**Results:**

In this study, we addressed the gut microbial antibiotic resistance genes profile using the metagenomic data from two online public pancreatic cancer cohorts. We found a high degree of data concordance between the two cohorts, which can therefore be used for cross-sectional comparisons. Meanwhile, we used two strategies to predict antibiotic resistance genes and compared the advantages and disadvantages of these two approaches. We also constructed microbe-antibiotic resistance gene networks and found that most of the hub nodes in the networks were antibiotic resistance genes.

**Conclusions:**

In summary, we describe the panorama of antibiotic resistance genes in the gut microbes of patients with pancreatic cancer. We hope that our study will provide new perspectives on treatment options for the disease.

## 1 Introduction

Pancreatic cancer, particularly pancreatic ductal adenocarcinoma (PDAC), is one of the deadliest cancer, with a 5-year overall survival rate of 11% (Siegel et al., 2022). The high lethality of PDAC is attributed to both late diagnosis and limited therapeutic options. This is because symptoms are often nonspecific and only become apparent in the advanced stages of the disease, when tumors may already be locally non-resectable or have metastasized. Surgery is the only potential curative treatment, but this method is only possible in the early stage. Combinatorial chemotherapy remains the standard of care for PDAC patients, but most patients present with an advanced disease characterized by both inherent and rapidly acquired chemoresistance to current anticancer treatments (Zeng et al., 2019).

In the past decade, microbiome studies have explored the association of pancreatic cancer with the human oral and gut microbiomes (Farrell et al., 2012; Ren et al., 2017; Fan et al., 2018; Matsukawa et al., 2021; Kartal et al., 2022). A study identified 30 gut and 18 oral species significantly linked to pancreatic cancer, exhibiting AUCs ranging from 0.78 to 0.82 (Nagata et al., 2022). In addition, epidemiological investigations have revealed a connection between periodontitis and an elevated risk of pancreatic cancer development (Michaud et al., 2007). Notably, *Neisseria elongata* and *Porphyromonas gingivalis* in saliva have been associated with an increased risk of pancreatic cancer development (Fan et al., 2018). Despite the pancreas not being part of the alimentary canal, the suggested translocation of microbiota from the gut to the pancreas is thought to occur through the sphincter of Oddi (Ansari et al., 2023). Studies using mouse models strongly suggested profound associations of gut microbiome with pancreatic cancer (Pushalkar et al., 2018; Thomas and Jobin, 2020). Moreover, studies suggested that microbiome ablation with antibiotics in mouse models improved tumor immune surveillance and improved responses to PD-1 blockade (Pushalkar et al., 2018). On the one hand, antibiotic use may lead to microbiota dysbiosis, potentially fostering chemoresistance and influencing treatment outcomes (Routy et al., 2018). On the other hand, much evidence suggested that the gut microbiota plays a role in determining resistance to various anticancer treatments, encompassing conventional chemotherapy, immunotherapy, radiotherapy, and surgery (Garajova et al., 2021). Several studies have highlighted the microbiome’s involvement in drug resistance within gastrointestinal cancers, including esophageal and pancreatic cancers (Yang et al., 2009; Wilkinson et al., 2018; Garajova et al., 2021). An increasing number of studies has shown that gut microbiota may impact resistance to commonly used anticancer drugs such as irinotecan, oxaliplatin, cyclophosphamide, 5-fluorouracil, gemcitabine, and anthracyclines (Bloemen et al., 2010; Iida et al., 2013; Sivan et al., 2015; Goubet et al., 2018). For instance, *Bacteroides* spp., residing in the gastrointestinal tract, expedite the conversion of sorivudine (a synthetic thymidine analogue used as an antiviral agent) into bromovinyluracil (BVU), an intermediate product that inhibits 5-FU degradation by the enzyme dihydropyrimidine dehydrogenase (Nakayama et al., 1997; Chae et al., 2020). However, the gut microbial antibiotic resistance genes profiling of pancreatic cancer patients has never reported compared to healthy cohort. Therefore, enhancing our understanding of the gut microbiota and its interactions with anticancer drugs will empower us to devise innovative treatment strategies, thereby contributing to treatments of cancer patients.

In this study, we addressed the gut microbial antibiotic resistance genes profile using the metagenomic data from online public pancreatic cancer cohorts. By studying the types, abundance, copy number, and affiliated microorganisms of antibiotic resistance genes in the gut microbes of pancreatic cancer patients, we describe the panorama of antibiotic resistance genes in the gut microbes of patients with the disease. We aimed to find out the main types of antibiotic resistance genes and the microorganisms. This will provide a theoretical basis for more accurate assessment of the patient’s condition and adjustment of the treatment plan for pancreatic cancer in the future.

## 2 Methods

### 2.1 Public data acquisition and quality control

All data in this study were obtained from the NCBI database (https://www.ncbi.nlm.nih.gov/) (**Supplementary Table S1**) and downloaded using fastq-dump. The total number of samples in our analysis is 202. The raw data were preprocessed using Trim_Galore (Version 0.4.5) to obtain the clean data. Since some of the metagenomic data had a high proportion of human DNA, we removed the human DNA from the clean data. The clean data were mapped to the reference sequence of human genome (hg38) using bwa (Li and Durbin, 2009), and the sam file was processed into sort.bam file by samtools (Li et al., 2009). The sort.bam file was converted into bed file by bamToBed (Quinlan and Hall, 2010). An in-house perl program for removing human DNA was used, and the samples that clean data over 800 megabases were used for subsequent analysis.

### 2.2 Antibiotic resistance gene identification

In this study, two strategies were used for the prediction of antibiotic resistance genes: CARD database mapping vs. genome assembly and RGI prediction. In the CARD database mapping strategy, the reference sequence of antibiotic resistance genes (https://card.mcmaster.ca/download) was downloaded from the Comprehensive Antibiotic Resistance Database (CARD) (Alcock et al., 2020). The index file of reference sequences was constructed by Salmon (Patro et al., 2017). The clean data after filtering human DNA were performed using Salmon to quantitative the abundance of antibiotic resistance genes (Patro et al., 2017). In the genome assembly and RGI (Resistance Gene Identifier) prediction strategy, first, the genome assembly were performed based on clean data using metaspades (Nurk et al., 2017). Then, the assembled genome sequences were annotated using the genome annotation software prokka (Seemann, 2014). Antibiotic resistance gene prediction was performed based on gene sequences using the RGI to obtain the copy number of the antibiotic resistance.

### 2.3 Taxonomy classification and other bioinformatics analysis

Kraken2 coupled with Bracken strategy were used for taxonomy annotations and abundance quantification based on clean data after filtering human DNA (Wood et al., 2019; Lu and Salzberg, 2020). The relationships between microbes and antibiotic resistance genes in network analysis were determined by genome assembly and RGI prediction strategy. The network between microbes and antibiotic resistance genes was performed by Cytoscape (Otasek et al., 2019). LEfSe was used for differential abundance microbial identification (Segata et al., 2011).

### 2.4 Statistical analysis

Analysis of differences between groups in this study was done by Wilcox test, and R script was used for statistical analysis. We consider p-values ≤ 0.05 to be significant. Ggplot2 package was used for plotting in this study.

## 3 Results

### 3.1 Overview of the cohorts and metagenomic data

Two pancreatic cancer cohorts sourced from two different studies were selected: patients of one cohort only collected from Japan between August 2014 and September 2019 (called Cohort 1) (Matsukawa et al., 2021) and the other cohort collected from a multinational study including Japan, Spain, and Germany (called Cohort 2) (Nagata et al., 2022). In addition, we also included a healthy cohort as a control, which is also sourced from the multinational study. Each cohort contained oral samples and fecal samples (see **Supplementary Table S1**). We pre-processed the downloaded raw data and removed the human DNA sequences, and we found that the proportion of the human DNA in the fecal samples was small, while the proportion of the human DNA in the oral cavity was large. The proportion of the human DNA in several oral samples was more than 95%, so we screened the samples, and we only retained samples whose file size were larger than 800 M for subsequent analyses. In sum, we obtained a total of 202 metagenomic samples in the three cohorts.

### 3.2 Quantitative analysis of microbial abundance in pancreatic cancer cohorts

**Figure 1:**
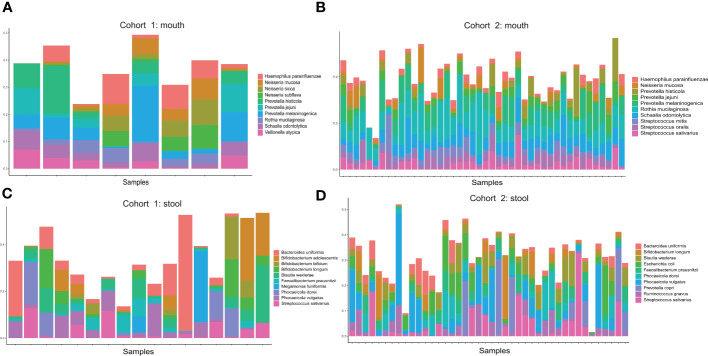
**(A)** Distribution of the top 10 microorganisms (species level) in abundance of oral samples from Cohort 1. **(B)** Distribution of the top 10 microorganisms (species level) in abundance of oral samples from Cohort 2. **(C)** Distribution of the top 10 microorganisms (species level) in abundance of stool samples from Cohort 1. **(D)** Distribution of the top 10 microorganisms (species level) in abundance of stool samples from Cohort 2.

We calculated the abundance of samples from two pancreatic cancer cohorts (oral and fecal) at the species level and show the top 10 species in abundance of each cohort. In the oral samples of Cohort I, which had only eight samples, three of the top 10 species were from the genus *Prevotella*, another three species from the genus *Neisseria*, and the others from different genus (**Figure 1A**). In the oral samples of Cohort II, three of the top 10 species were from the genus *Prevotella*, and these three species were also present in the oral samples of Cohort I at the same time (**Figure 1B**). In addition, another three species from the genus *Streptococcus* were also present in the top 10 species of the cohort II. It is worth noting that 7 of the top 10 species overlapped in both oral cohorts, indicating better reproducibility of the data from these two cohorts. In fecal samples, Cohort I contained a total of 17 samples. The top 10 species were mainly from genus *Bifidobacterium*, genus *Phocaeicola*, *Faecalibacterium prausnitzii*, *Streptococcus salivarius*, and *Bacteroides uniformis* (**Figure 1C**). In Cohort II, the top 10 species in the fecal sample included *Bifidobacterium longum*, *Streptococcus salivarius*, and *Faecalibacterium prausnitzii* (**Figure 1D**). Coincidentally, there are also seven shared species of top 10 species in abundance for both cohorts of fecal samples. We also show the top 10 species for both cohorts (oral samples and fecal samples) based on heatmaps. The difference in the abundance of the top 10 species between the disease and control groups is not obvious (**Supplementary Figure S1**). Furthermore, we compared the top 300 species in microbial abundance for the oral and fecal samples, and we found that the two cohorts overlapped by 85% for both oral and fecal (85% for oral and 86% for fecal), which shows that the two population cohorts are highly reproducible (**Supplementary Figure S2**). We also found *Streptococcus salivarius* to be present in both fecal cohorts and in the oral cohort of Cohort II.

### 3.3 Identifying antibiotic resistance genes in pancreatic cancer cohorts

**Figure 2:**
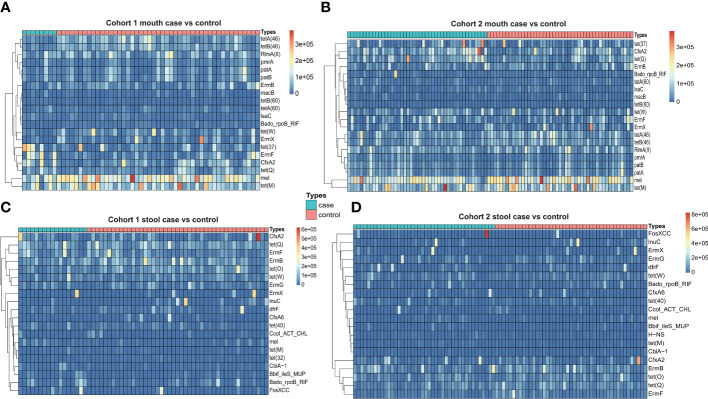
**(A)** Heatmap of antibiotic resistance gene abundance of oral samples from Cohort 1 (case vs. control). **(B)** Heatmap of antibiotic resistance gene abundance of oral samples from Cohort 2 (case vs. control). **(C)** Heatmap of antibiotic resistance gene abundance of stool samples from Cohort 1 (case vs. control). **(D)** Heatmap of antibiotic resistance gene abundance of stool samples from Cohort 2 (case vs. control).

To systematically identify antibiotic resistance genes, we used two strategies for prediction: CARD database mapping vs. genome assembly and RGI prediction. Utilizing the mapping strategy of the CARD database, which relies on the reference dataset of antibiotic resistance genes offered by the CARD database, we aligned the clean reads with the reference sequences. Then, we calculated the abundance and frequency of antibiotic resistance genes in oral and fecal samples from the two cohorts separately. In addition, we also added an extra healthy cohort with oral and fecal samples as control. In the pancreatic cancer cohorts, we identified a total of 130 types of antibiotic resistance genes in oral samples. The top 10 most frequent genes were mostly from tetracyclines, with an average of 36 types of antibiotic resistance genes detected per sample. We selected the top 20 antibiotic resistance genes in terms of abundance for comparison between different cohorts of pancreatic cancer and healthy individuals, and we found that the abundance and the frequency of *tet(M)* and *mel* genes were higher overall. The Wilcox test showed that the abundance of antibiotic resistance genes in oral samples of pancreatic cancer were not significantly different from that of healthy individuals (**Figures 2A, B**). We then analyzed fecal samples and identified a total of 321 types of antibiotic resistance genes, with most of the top 10 genes with the highest frequency also coming from the tetracyclines but differing from the typing of antibiotic resistance genes in the oral samples, with an average of 82 types of antibiotic resistance genes detected per sample. For fecal samples, we also selected the top 20 antibiotic resistance genes in terms of abundance for comparison between samples from different cohorts of pancreatic cancer and healthy individuals. The abundance of antibiotic resistance genes in fecal samples were relatively stable, and the Wilcox test did not reveal any difference in the abundance of fecal antibiotic resistance genes between pancreatic cancer samples and those from healthy individuals (**Figures 2C, D**).

**Figure 3:**
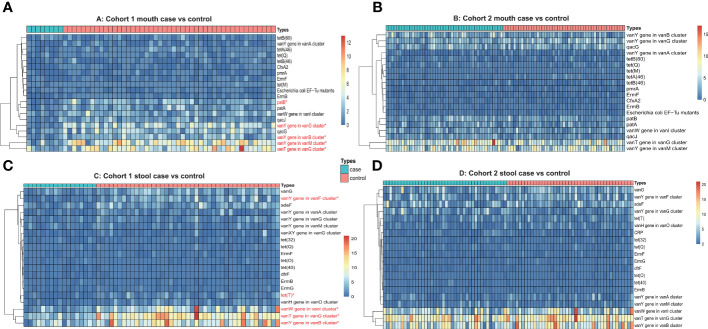
**(A)** Heatmap of antibiotic resistance gene copy number of oral samples from Cohort 1 (case vs. control). **(B)** Heatmap of antibiotic resistance gene copy number of oral samples from Cohort 2 (case vs. control). **(C)** Heatmap of antibiotic resistance gene copy number of stool samples from Cohort 1 (case vs. control). **(D)** Heatmap of antibiotic resistance gene copy number of stool samples from Cohort 2 (case vs. control). The red font and * means p ≤0.05.

In terms of genome assembly and RGI prediction strategy, we first obtained the gene sequences of each sample by metagenomic assembly and then predicted the copy number of antibiotic resistance genes by rgi software. Similar to the previous strategy, we also calculated the frequency of antibiotic resistance genes in oral and fecal samples from each of the three cohorts (two pancreatic cancer cohorts and one healthy cohort). In the pancreatic cancer oral samples, we identified a total of 72 types of antibiotic resistance genes, with an average of 39 types of antibiotic resistance genes per sample, and half of the top 10 most frequent genes were vancomycin resistance genes. We also selected the top 20 antibiotic resistance genes in terms of copy number for comparison between different cohorts of pancreatic cancer and healthy individuals. The Wilcox test showed significant differences in the copy number of *vanT*, *vanY*, and *patB* genes in different gene clusters of the pancreatic cancer cohort I compared with the healthy cohort (**Figures 3A, B**). In pancreatic cancer stool samples, we identified a total of 227 types of antibiotic resistance genes, with an average of 73 types of antibiotic resistance genes per sample, and half of the 10 genes with the highest frequency also belonged to vancomycin-resistant genes, and three genes belonged to tetracycline-resistant genes. In a cross-sectional cohort’s comparison of the top 20 copy number antibiotic resistance genes, four vancomycin-resistant genes and one tetracycline-resistant gene showed significant copy number differences between the pancreatic cancer Cohort I and the healthy cohort (**Figures 3C, D**).

### 3.4 Antibiotic resistance genes and their microbes

To figure out the affiliations between antibiotic resistance genes and gut microbes, we constructed a network of antibiotic resistance genes interacting with microbes. We constructed the network based on antibiotic resistance genes obtained from genome assembly and RGI prediction because we considered that this strategy provides more accurate links. Furthermore, we used only two cohorts of pancreatic cancer patients (oral samples and intestinal samples). We performed the network construction according to the oral samples and the fecal samples, respectively, and merged the connections of the two cohorts (**Supplementary Figure S3**). We found a total of 283 non-redundant connections in the antibiotic resistance genes–microbes interaction network of the oral samples, of which nine of the hub nodes in the top 10 of the degree rank belonged to antibiotic resistance genes, and most of them were from vancomycin-resistant genes (the top 3 hub nodes in order were *vanT* gene in *vanG* cluster, 67 links; *vanY* gene in *vanM* cluster, 27 links; *vanY* gene in *vanB* cluster, 25 links), and the other top 10 hub nodes was species *Streptococcus* sp. (11 links) (**Supplementary Figure 3A**). We analyzed the affiliations of the top 9 hub antibiotic resistance genes, which came from a total of 59 genera, with the top 5 genera being *Streptococcus* (64), *Veillonella* (20), *Prevotella* (20), *Bacteroides* (8), and *Neisseria* (7). From microbiological perspective, the overall average degree of microbes was only 2, and most of the microbes with a degree of more than 5 were from the genera *Streptococcus* and *Prevotella*. In the antibiotic resistance genes–microbes network of fecal samples, there were a total of 556 non-redundant connections, of which the top 10 hub nodes were all antibiotic resistance genes, and nine were from vancomycin-resistant genes (the top 3 hub nodes in order were *vanT* gene in *vanG* cluster, 134 links; *vanW* gene in *vanI* cluster, 70 links; *vanY* gene in *vanB* cluster, 62 links) (**Supplementary Figure 3B**). We also analyzed the affiliations of the top 10 hub antibiotic resistance genes, which came from a total of 136 genera, with the top 5 genera being *Streptococcus* (33), *Blautia* (27), *Ruminococcus* (25), *Bacteroides* (23), and *Faecalibacterium* (15). Taking the microbiological point, the overall average degree of microbes was 2. A total of 30 microbes with a degree of more than 5, scattered among different genera, and no dominant genus was found.

### 3.5 Antibiotic resistance genes in differential abundant microbiotas

Differential abundant microbes between groups are often associated with disease and health. We used LEfSe to identify differential abundant microbes in two pancreatic cancer cohorts versus healthy control (**Supplementary Figure S4**). In oral samples, we identified 31 and 33 differential abundant microbes in the two cohorts, respectively. In fecal samples, we identified 48 and 55 differential abundant microbes in the two cohorts, respectively. In this study, we focused on the antibiotic resistance genes of the differential abundant microbes that were significantly enriched in the cohorts of pancreatic cancer patients (combined the differential abundant microbes from the two cohorts). In the oral samples, a total of 25 differential abundant microbes were significantly enriched in the pancreatic cancer cohorts. Integrating the results of the antibiotic resistance genes described above, we found that 11 of these differential abundant microbes with eight types of antibiotic resistance genes (**Supplementary Table S2**), and the 11 differential abundant microbes were mainly from the genus *Prevotella* (including six species), while the others were *Fusobacterium pseudoperiodonticum* and *Bacteroides heparinolyticus*. The number of linkages of vancomycin-resistant genes was the highest in eight types of antibiotic resistance genes, and the *vanT* gene in *vanG* cluster had the most connections in the vancomycin-resistant genes. In the fecal samples, integrating the results of a total of 29 differential abundant microbes that were significantly enriched in the pancreatic cancer cohorts and the antibiotic resistance genes mentioned above, we found that 12 differential abundant microbes with seven types of antibiotic resistance genes (**Supplementary Table S3**). Most of 12 differential abundant microbes came from *Streptococcus* and *Klebsiella*. The number of vancomycin-resistant genes in the seven types of antibiotics was also the highest, but the genotypes were different from the oral samples. The antibiotic resistance genes in the fecal samples were mainly *vanG*, *vanT*, *vanW*, and *vanY*.

## 4 Discussion

Antibiotic resistance has been a globally health concern. It affects the therapeutic efficacy of diseases. Traditional methods of antibiotic resistance testing are mainly based on minimal inhibitory concentration (MIC) test of cultured isolates of microorganisms. The gut contains thousands of microbial species, most of which are uncultured or difficult to culture. It is difficult to detect gut antibiotic resistance using traditional methods because of their large microbial community. Moreover, detecting antibiotic resistance of isolates does not reflect the overall of antibiotic resistance in gut microbes. The whole genome sequencing-based metagenomic technology can detect most of the DNA fragments in one sample and thus can detect the antibiotic resistance genes from gut. It can reveal the overall of antibiotic drug resistance to a certain extent by this method. Pancreatic cancer is one of the common malignant tumors in the digestive tract and is known as the “king of cancers” in the field of oncology. Studies in this field of microbiology have also been widely reported, but the gut microbial antibiotic resistance profile of pancreatic cancer are rarely reported. Here, we investigated the gut microbial antibiotic resistance profiles of pancreatic cancer using two online public data cohorts with oral and fecal samples, which can be used to validate the results against each other. In addition, we included a cohort of healthy individuals that also contained oral and fecal samples as a control. We found that the two pancreatic cancer cohorts had an overlap of more than 85% in abundance of top 300 species for both oral and fecal samples, suggesting that the two cohorts were well reproducible and could be used for validation of the results in subsequent analyses.

While numerous anticancer therapeutic regimens have achieved clinical success, the enduring challenges of heterogeneous response and resistance to chemotherapy and immunotherapy remain the hallmarks of cancer therapy. Recent findings have unveiled a correlation between the microbiota and the development of chemoresistance (Choy et al., 2018). Hence, integrating microbiome-modulating regimens (such as antibiotics, probiotics, and dietary interventions) with anticancer treatment could offer innovative therapeutic approaches for cancers associated with dysbiosis. Several studies have indicated the significant impact of gut microbiota on drug metabolism and encompassing anticancer drugs (Ma et al., 2019). This process may result in heightening or diminishing drug activity, coupled with variations in toxicity levels. Consequently, manipulating the microbial network through interventions like fecal transplantation or probiotics emerges as a promising strategy that may enhance the treatment efficacy for cancer patients. Ongoing clinical trials are exploring the potential of the microbiome to enhance the management of pancreatic cancer. In our study, genera such as *Prevotella* and *Fusobacterium* were significantly enriched in the pancreatic cancer cohort, compared with healthy cohort in oral samples. Based on a recent study about oral microbiota in pancreatic cancer, patients had a higher amount of *Prevotella* compared with the healthy control group (Wei et al., 2020). Fan et al. found that *Fusobacterium* and several bacteria might be a protective factor in oral samples of pancreatic cancer (Fan et al., 2018). Previous studies have reported that vancomycin-resistant genes (*vanT* gene and *vanY* gene) were identified from *Streptococcus*, *Veillonella*, and *Prevotella*. Kwack et al. revealed the biological mechanisms of vancomycin tolerance in the oral commensal bacterium *Streptococcus anginosus* by whole genome and RNA sequencing (Kwack et al., 2022). Li et al. tested the vancomycin resistance in *Veillonella* strains from oral of healthy adults by agar dilution method (Li et al., 2022). In the fecal samples, *Prevotella*, *Streptococcus*, and *Klebsiella* were the differential abundant microbes in the pancreatic cancer cohort compared with healthy cohort (Kabwe et al., 2022). The genus *Streptococcus* has been studied for its association with cancer progression and treatment outcomes over an extended period (Sobocki et al., 2021). Matsukawa et al. found that *Klebsiella*, *Streptococcus*, and other several microbiotas can be treated as prognostic factors for pancreatic cancer (Matsukawa et al., 2021). In fecal samples of pancreatic cancer, vancomycin-resistant genes (*vanT* gene, *vanW* gene, and *vanY* gene) were also found in some popular gut microbes (such as *Streptococcus*, *Blautia*, and *Ruminococcus*) (Stogios and Savchenko, 2020). An earlier study showed that *vanD* and *vanG*-Like gene clusters were located in *Ruminococcus* species, which isolated from human bowel flora (Domingo et al., 2007). The *vanB* gene has also been found in a vancomycin-resistant isolate of *Streptococcus bovis* isolated from a stool swab (Poyart et al., 1997). The above findings suggest the importance of vancomycin-resistant microbial communities in pancreatic cancer.

Two strategies are currently used to study the antibiotic resistance gene profiles of gut microbes: CARD database mapping vs. genome assembly and RGI prediction. Both strategies have their advantages: CARD database mapping strategy is more sensitive and can detect more antibiotic resistance genes, which is mainly used to calculate the abundance of those genes. The disadvantages of this strategy are that it may be affected by the length of the reads, which leads to a low prediction accuracy, and the range of antibiotic resistance genes detected may be limited by the content of the database. Another method for antibiotic resistance gene prediction is genome assembly and RGI prediction, which is a strategy based on the annotation of assembled genome sequences and is mainly used to detect the presence or absence of antibiotic resistance genes and the copy numbers. It offers the benefit of relatively high accuracy and the potential for uncovering new antibiotic resistance genes. However, a drawback is the inability to quantify their abundance. In this study, we compared these two prediction strategies. It was found that CARD database mapping strategy detected more types of antibiotic resistance genes than genome assembly and RGI prediction in both oral and fecal samples. In terms of the average number of antibiotic resistance genes of each sample, the two strategies did not differ much in oral and fecal samples. In terms of frequency, for both strategies, the genes with high frequency in oral and fecal samples were from the vancomycin and tetracycline resistance gene families, which shows that the results predicted by the two methods are in good agreement. However, there are some differences between the two strategies. According to the results of the heatmap and the Wilcox test, genome assembly and RGI prediction identified significant differences in some vancomycin-resistant genes between the pancreatic cancer patient cohort and the healthy cohort. However, this distinction was not observed with the CARD database mapping strategy. In summary, we found that the CARD database mapping strategy was superior in sensitivity in the prediction of the types of antibiotic resistance genes, whereas genome assembly and RGI prediction was better able to detect anomalies in copy number between groups.

There are still some shortcomings in this study. First, because we used publicly available online data, we lacked information on oral and fecal pairings; thus, we were unable to compare the antibiotic resistance genes between oral and fecal samples in a single individual. Second, the online public data also lacked information on antibiotic use and treatment of the patients, so we were unable to map antibiotic resistance genes to patients’ clinical information. Finally, the resistance of some microbes requires isolation by bacteria culture and measure by MIC. We hope that future research may complement the shortcomings.

## 5 Conclusion

We conducted an extensive evaluation of antibiotic resistance genes using both oral and fecal samples sourcing from two publicly available online datasets of pancreatic cancer populations. We found a high degree of data concordance between the two cohorts, which can therefore be used for cross-sectional comparisons. Meanwhile, we used two strategies to predict antibiotic resistance genes and compared the advantages and disadvantages of these two approaches. Based on genome assembly and RGI prediction strategy, we found that four vancomycin-resistant genes and one tetracycline-resistant gene differed significantly in copy number between the pancreatic cancer Cohort I and the healthy cohort. We also constructed microbe-antibiotic resistance gene networks and found that most of the hub nodes in the networks were antibiotic resistance genes. We hope that our research will contribute to adjuvant therapy for pancreatic cancer patients by designing new antibiotic regimens that can be targeted to alter the abundance of dysbiotic microbiota in the future.

## Data availability statement

The datasets presented in this study can be found in online repositories. The names of the repository/repositories and accession number(s) can be found in the article/**Supplementary Material**.

## Ethics statement

The studies involving humans were approved by PUMCH Institutional Review Board. The studies were conducted in accordance with the local legislation and institutional requirements. Written informed consent for participation was not required from the participants or the participants’ legal guardians/next of kin in accordance with the national legislation and institutional requirements.

## Author contributions

XL: Data curation, Formal analysis, Investigation, Methodology, Validation, Visualization, Writing – original draft, Writing – review & editing. KL: Conceptualization, Data curation, Formal analysis, Investigation, Methodology, Resources, Software, Validation, Visualization, Writing – original draft, Writing – review & editing. YY: Formal analysis, Methodology, Resources, Software, Writing – original draft. DC: Methodology, Project administration, Software, Supervision, Writing – original draft. XX: Formal analysis, Investigation, Methodology, Resources, Supervision, Writing – review & editing. ZH: Data curation, Investigation, Project administration, Resources, Software, Supervision, Visualization, Writing – original draft, Writing – review & editing. WW: Data curation, Funding acquisition, Investigation, Project administration, Resources, Supervision, Writing – original draft, Writing – review & editing.

## Supplementary material

Supplementary Figure 1 **(A)** Heatmap of top 10 species abundance of oral samples from Cohort 1 (Case vs Control). **(B)** Heatmap of top 10 species abundance of oral samples from Cohort 2 (Case vs Control). **(C)** Heatmap of top 10 species abundance of stool samples from Cohort 1 (Case vs Control). **(D)** Heatmap of top 10 species abundance of stool samples from Cohort 2 (Case vs Control).

Supplementary Figure 2 **(A)** The venn diagrams of top 300 microbial abundances in oral samples from different cohorts. **(B)** The venn diagrams of top 300 microbial abundances in stool samples from different cohorts.

Supplementary Figure 3 **(A)** Network between microbes and antibiotic resistance genes network from oral samples in two cohorts. **(B)** Network between microbes and antibiotic resistance genes from stool samples in two cohorts. Red nodes represent antibiotic resistance genes and light-blue nodes represent microorganisms.

Supplementary Figure 4 **(A)** Lefse analysis at the species level between control and case oral samples from cohort 1. **(B)** Lefse analysis at the species level between control and case oral samples from cohort 2. **(C)** Lefse analysis at the species level between control and case stool samples from cohort 1. **(D)** Lefse analysis at the species level between control and case stool samples from cohort 2.
