# Supplementary material for: Gut resistome profiling reveals high diversity and fluctuations in pancreatic cancer cohorts
Source: Front Cell Infect Microbiol. 2024 Feb 7;14:1354234. doi: 10.3389/fcimb.2024.1354234 (PMC10879602; doi:10.3389/fcimb.2024.1354234)
Supplement: Supplementary Figure 1 — (A) Heatmap of top 10 species abundance of oral samples from Cohort 1 (Case vs Control). (B) Heatmap of top 10 species abundance of oral samples from Cohort 2 (Case vs Control). (C) Heatmap of top 10 species abundance of stool samples from Cohort 1 (Case vs Control). (D) Heatmap of top 10 species abundance of stool samples from Cohort 2 (Case vs Control). [file DataSheet_1.pdf]

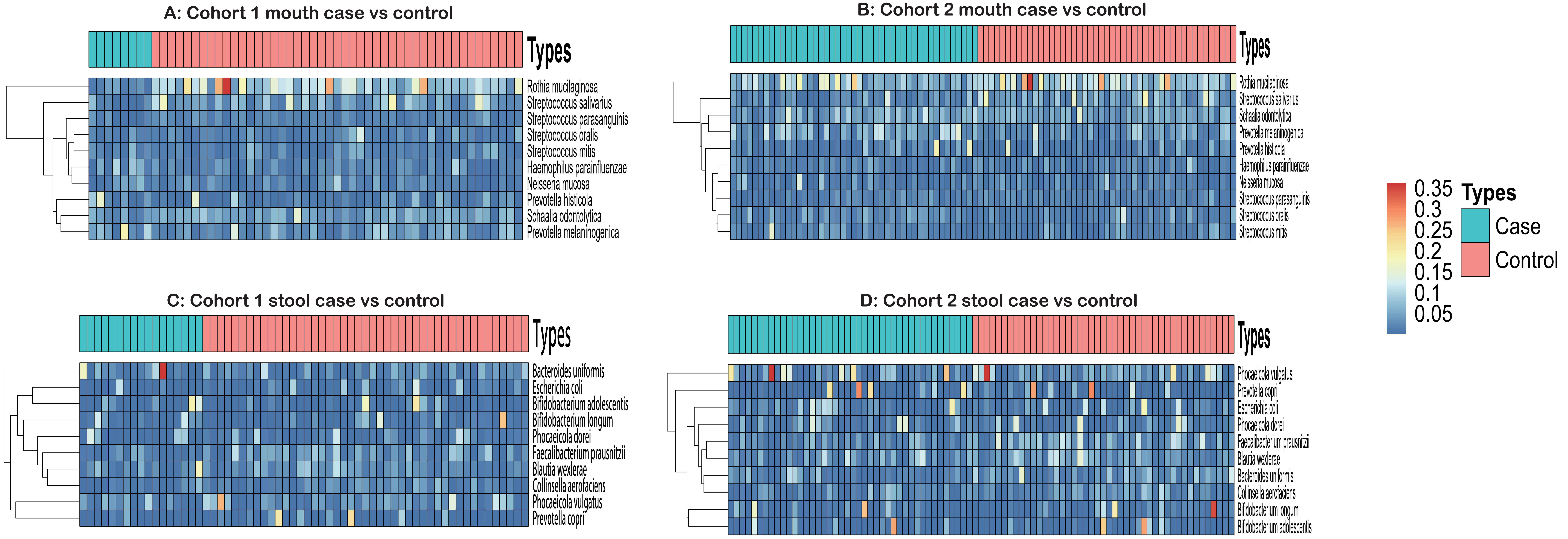
A: Cohort 1 mouth case vs control
Types
Rothia mucilaginosa
Streptococcus salivarius
Streptococcus parasanguinis
Streptococcus oralis
Streptococcus mitis
Haemophilus parainfluenzae
Neisseria mucosa
Prevotella histicola
Schaalia odontolytica
Prevotella melaninogenica
B: Cohort 2 mouth case vs control
Types
Rothia mucilaginosa
Streptococcus salivarius
Schaalia odontolytica
Prevotella melaninogenica
Prevotella histicola
Haemophilus parainfluenzae
Neisseria mucosa
Streptococcus parasanguinis
Streptococcus oralis
Streptococcus mitis
C: Cohort 1 stool case vs control
Types
Bacteroides uniformis
Escherichia coli
Bifidobacterium adolescentis
Bifidobacterium longum
Phocaeicola dorei
Faecalibacterium prausnitzii
Blautia wexlerae
Collinsella aerofaciens
Phocaeicola vulgatus
Prevotella copri
D: Cohort 2 stool case vs control
Types
Phocaeicola vulgatus
Prevotella copri
Escherichia coli
Phocaeicola dorei
Faecalibacterium prausnitzii
Blautia wexlerae
Bacteroides uniformis
Collinsella aerofaciens
Bifidobacterium longum
Bifidobacterium adolescentis
0.35
0.3
0.25
0.2
0.15
0.1
0.05
Types
Case
Control
