# Supplementary figures and images for: Gut resistome profiling reveals high diversity and fluctuations in pancreatic cancer cohorts
Source: Front Cell Infect Microbiol. 2024 Feb 7;14:1354234. doi: 10.3389/fcimb.2024.1354234 (PMC10879602; doi:10.3389/fcimb.2024.1354234)

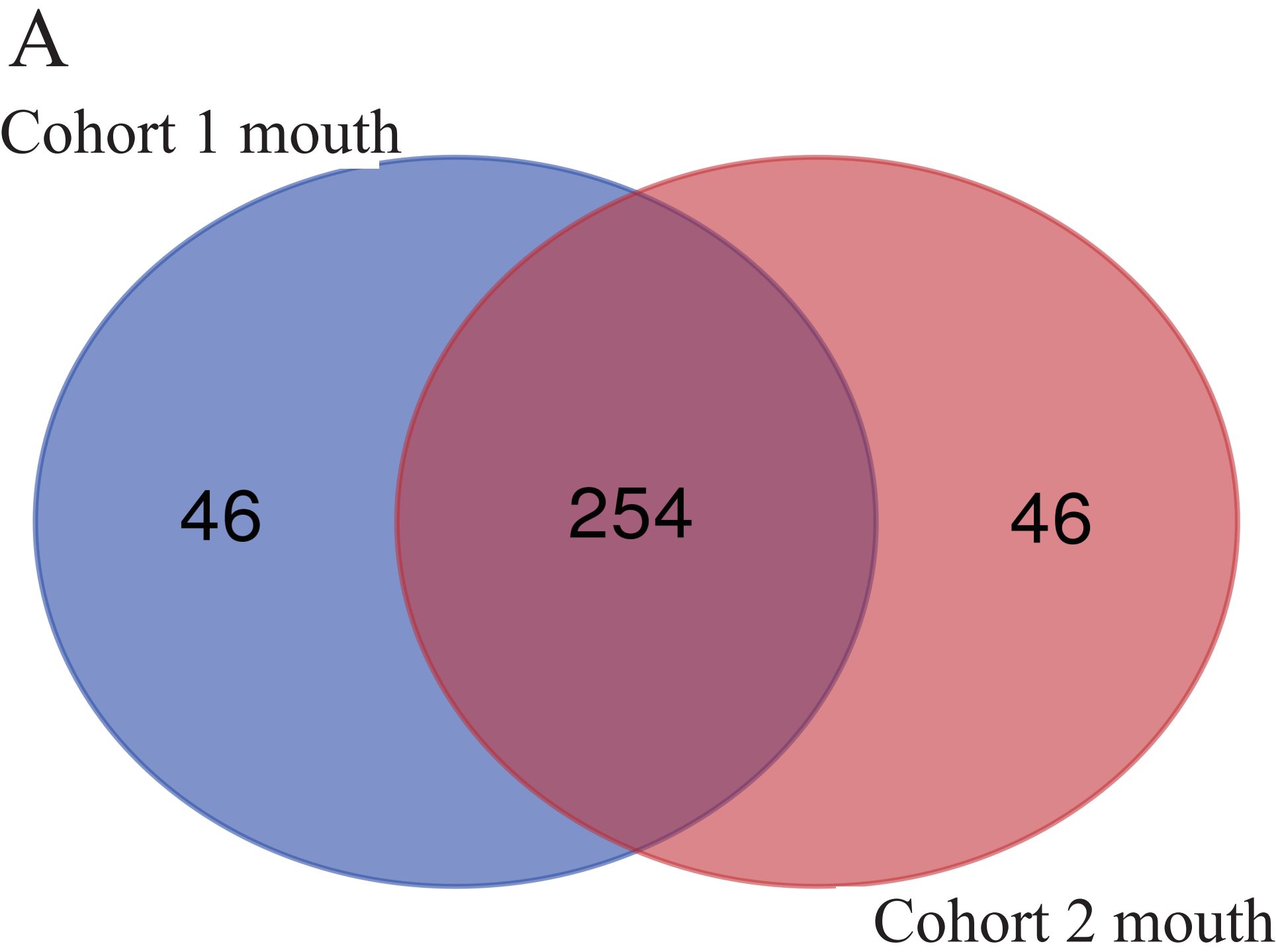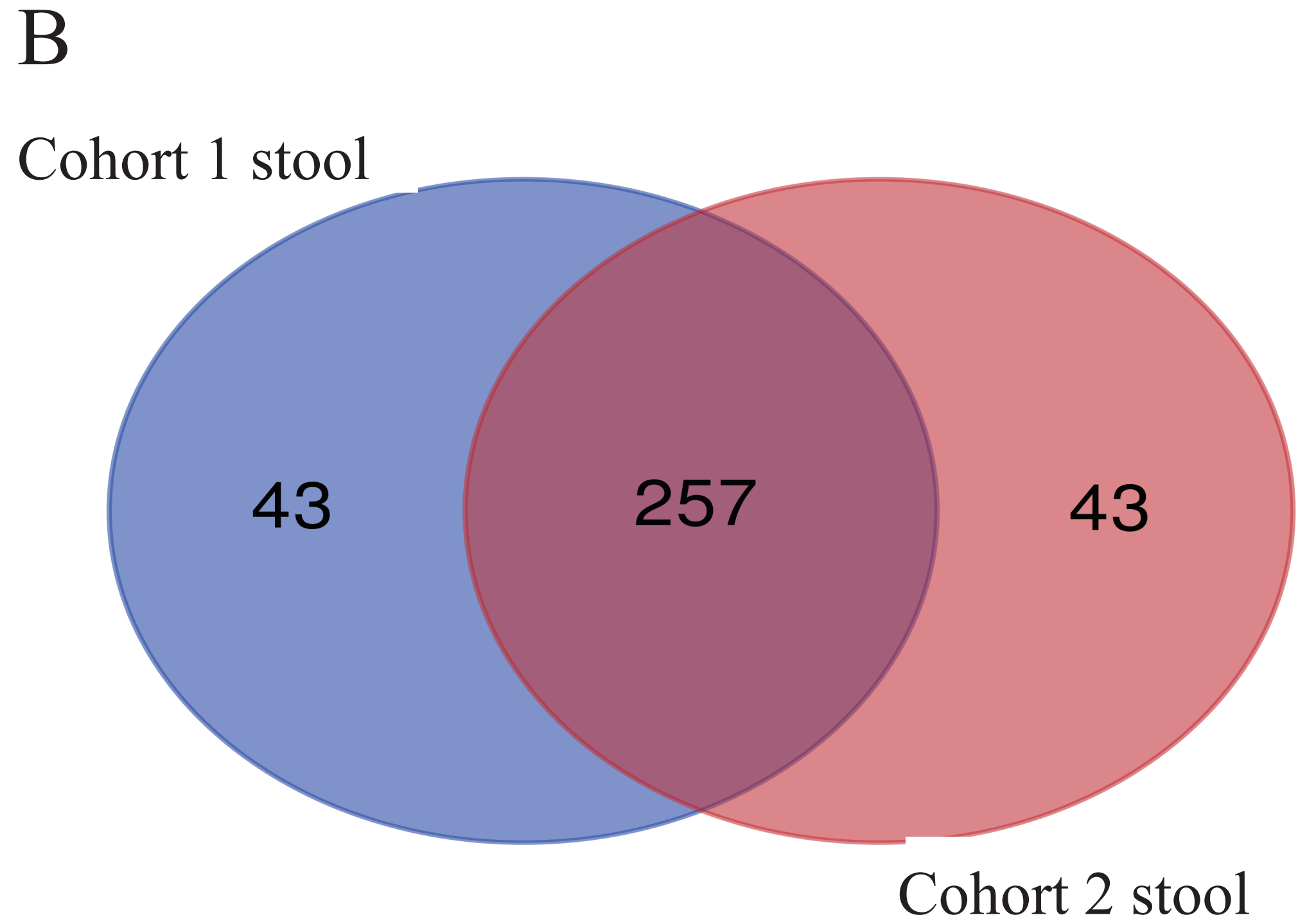

Supplement: Supplementary Figure 2 — (A) The venn diagrams of top 300 microbial abundances in oral samples from different cohorts. (B) The venn diagrams of top 300 microbial abundances in stool samples from different cohorts. [file DataSheet_2.pdf]
