# Supplementary material for: Gut resistome profiling reveals high diversity and fluctuations in pancreatic cancer cohorts
Source: Front Cell Infect Microbiol. 2024 Feb 7;14:1354234. doi: 10.3389/fcimb.2024.1354234 (PMC10879602; doi:10.3389/fcimb.2024.1354234)
Supplement: Supplementary Figure 4 — (A) Lefse analysis at the species level between control and case oral samples from cohort 1. (B) Lefse analysis at the species level between control and case oral samples from cohort 2. (C) Lefse analysis at the species level between control and case stool samples from cohort 1. (D) Lefse analysis at the species level between control and case stool samples from cohort 2. [file DataSheet_4.pdf]

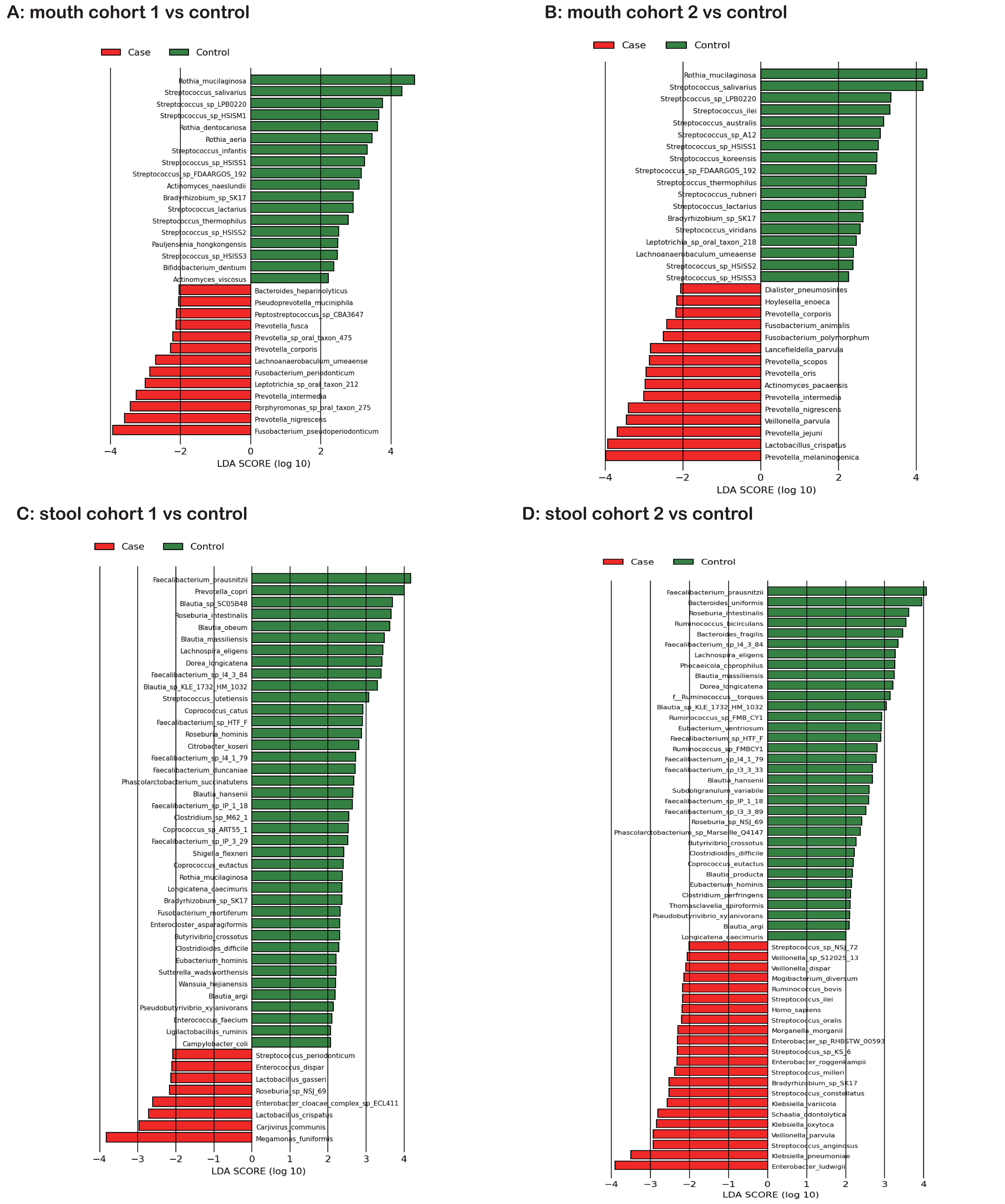

A: mouth cohort 1 vs control
Case
Control
Rothia_mucilaginosa
Streptococcus_salivarius
Streptococcus_sp_LPB0220
Streptococcus_sp_HSISM1
Rothia_dentocariosa
Rothia_aeria
Streptococcus_infantis
Streptococcus_sp_HSISS1
Streptococcus_sp_FDAARGOS_192
Actinomyces_naeslundii
Bradyrhizobium_sp_SK17
Streptococcus_lactarius
Streptococcus_thermophilus
Streptococcus_sp_HSISS2
Pauljensenia_hongkongensis
Streptococcus_sp_HSISS3
Bifidobacterium_dentium
Actinomyces_viscosus
Bacteroides_heparinolyticus
Pseudoprevotella_muciniphila
Peptostreptococcus_sp_CBA3647
Prevotella_fusca
Prevotella_sp_oral_taxon_475
Prevotella_corporis
Lachnoanaerobaculum_umeaense
Fusobacterium_periodonticum
Leptotrichia_sp_oral_taxon_212
Prevotella_intermedia
Porphyromonas_sp_oral_taxon_275
Prevotella_nigrescens
Fusobacterium_pseudoperiodonticum
−4
−2
0
2
4
LDA SCORE (log 10)
B: mouth cohort 2 vs control
Case
Control
Rothia_mucilaginosa
Streptococcus_salivarius
Streptococcus_sp_LPB0220
Streptococcus_ilei
Streptococcus_australis
Streptococcus_sp_A12
Streptococcus_sp_HSISS1
Streptococcus_koreensis
Streptococcus_sp_FDAARGOS_192
Streptococcus_thermophilus
Streptococcus_rubneri
Streptococcus_lactarius
Bradyrhizobium_sp_SK17
Streptococcus_viridans
Leptotrichia_sp_oral_taxon_218
Lachnoanaerobaculum_umeaense
Streptococcus_sp_HSISS2
Streptococcus_sp_HSISS3
Dialister_pneumosintes
Hoylesella_enoeca
Prevotella_corporis
Fusobacterium_animalis
Fusobacterium_polymorphum
Lancefieldella_parvula
Prevotella_scopos
Prevotella_oris
Actinomyces_pacaensis
Prevotella_intermedia
Prevotella_nigrescens
Veillonella_parvula
Prevotella_jejuni
Lactobacillus_crispatus
Prevotella_melaninogenica
−4
−2
0
2
4
LDA SCORE (log 10)
C: stool cohort 1 vs control
Case
Control
Faecalibacterium_prausnitzii
Prevotella_copri
Blautia_sp_SC05B48
Roseburia_intestinalis
Blautia_obeum
Blautia_massiliensis
Lachnospira_eligens
Dorea_longicatena
Faecalibacterium_sp_I4_3_84
Blautia_sp_KLE_1732_HM_1032
Streptococcus_lutetiensis
Coprococcus_catus
Faecalibacterium_sp_HTF_F
Roseburia_hominis
Citrobacter_koseri
Faecalibacterium_sp_I4_1_79
Faecalibacterium_duncaniae
Phascolarctobacterium_succinatutens
Blautia_hansenii
Faecalibacterium_sp_IP_1_18
Clostridium_sp_M62_1
Coprococcus_sp_ART55_1
Faecalibacterium_sp_IP_3_29
Shigella_flexneri
Coprococcus_eutactus
Rothia_mucilaginosa
Longicatena_caecimuris
Bradyrhizobium_sp_SK17
Fusobacterium_mortiferum
Enterocloster_asparagiformis
Butyrivibrio_crossotus
Clostridioides_difficile
Eubacterium_hominis
Sutterella_wadsworthensis
Wansuia_hejianensis
Blautia_argi
Pseudobutyrivibrio_xylanivorans
Enterococcus_faecium
Ligilactobacillus_ruminis
Campylobacter_coli
Streptococcus_periodonticum
Enterococcus_dispar
Lactobacillus_gasseri
Roseburia_sp_NSJ_69
Enterobacter_cloacae_complex_sp_ECL411
Lactobacillus_crispatus
Carjivirus_communis
Megamonas_funiformis
−4
−3
−2
−1
0
1
2
3
4
LDA SCORE (log 10)
D: stool cohort 2 vs control
Case
Control
Faecalibacterium_prausnitzii
Bacteroides_uniformis
Roseburia_intestinalis
Ruminococcus_bicirculans
Bacteroides_fragilis
Faecalibacterium_sp_I4_3_84
Lachnospira_eligens
Phocaeicola_coprophilus
Blautia_massiliensis
Dorea_longicatena
f__Ruminococcus__torques
Blautia_sp_KLE_1732_HM_1032
Ruminococcus_sp_FMB_CY1
Eubacterium_ventriosum
Faecalibacterium_sp_HTF_F
Ruminococcus_sp_FMBCY1
Faecalibacterium_sp_I4_1_79
Faecalibacterium_sp_I3_3_33
Blautia_hansenii
Subdoligranulum_variabile
Faecalibacterium_sp_IP_1_18
Faecalibacterium_sp_I3_3_89
Roseburia_sp_NSJ_69
Phascolarctobacterium_sp_Marseille_Q4147
Butyrivibrio_crossotus
Clostridioides_difficile
Coprococcus_eutactus
Blautia_producta
Eubacterium_hominis
Clostridium_perfringens
Thomasclavelia_spiroformis
Pseudobutyrivibrio_xylanivorans
Blautia_argi
Longicatena_caecimuris
Streptococcus_sp_NSJ_72
Veillonella_sp_S12025_13
Veillonella_dispar
Mogibacterium_diversum
Ruminococcus_bovis
Streptococcus_ilei
Homo_sapiens
Streptococcus_oralis
Morganella_morganii
Enterobacter_sp_RHBSTW_00593
Streptococcus_sp_KS_6
Enterobacter_roggenkampii
Streptococcus_milleri
Bradyrhizobium_sp_SK17
Streptococcus_constellatus
Klebsiella_variicola
Schaalia_odontolytica
Klebsiella_oxytoca
Veillonella_parvula
Streptococcus_anginosus
Klebsiella_pneumoniae
Enterobacter_ludwigii
−4
−3
−2
−1
0
1
2
3
4
LDA SCORE (log 10)
