# Supplementary material for: Gut resistome profiling reveals high diversity and fluctuations in pancreatic cancer cohorts
Source: Front Cell Infect Microbiol. 2024 Feb 7;14:1354234. doi: 10.3389/fcimb.2024.1354234 (PMC10879602; doi:10.3389/fcimb.2024.1354234)
Supplement: Supplementary Figure 3 — (A) Network between microbes and antibiotic resistance genes network from oral samples in two cohorts. (B) Network between microbes and antibiotic resistance genes from stool samples in two cohorts. Red nodes represent antibiotic resistance genes and light-blue nodes represent microorganisms. [file DataSheet_3.pdf]

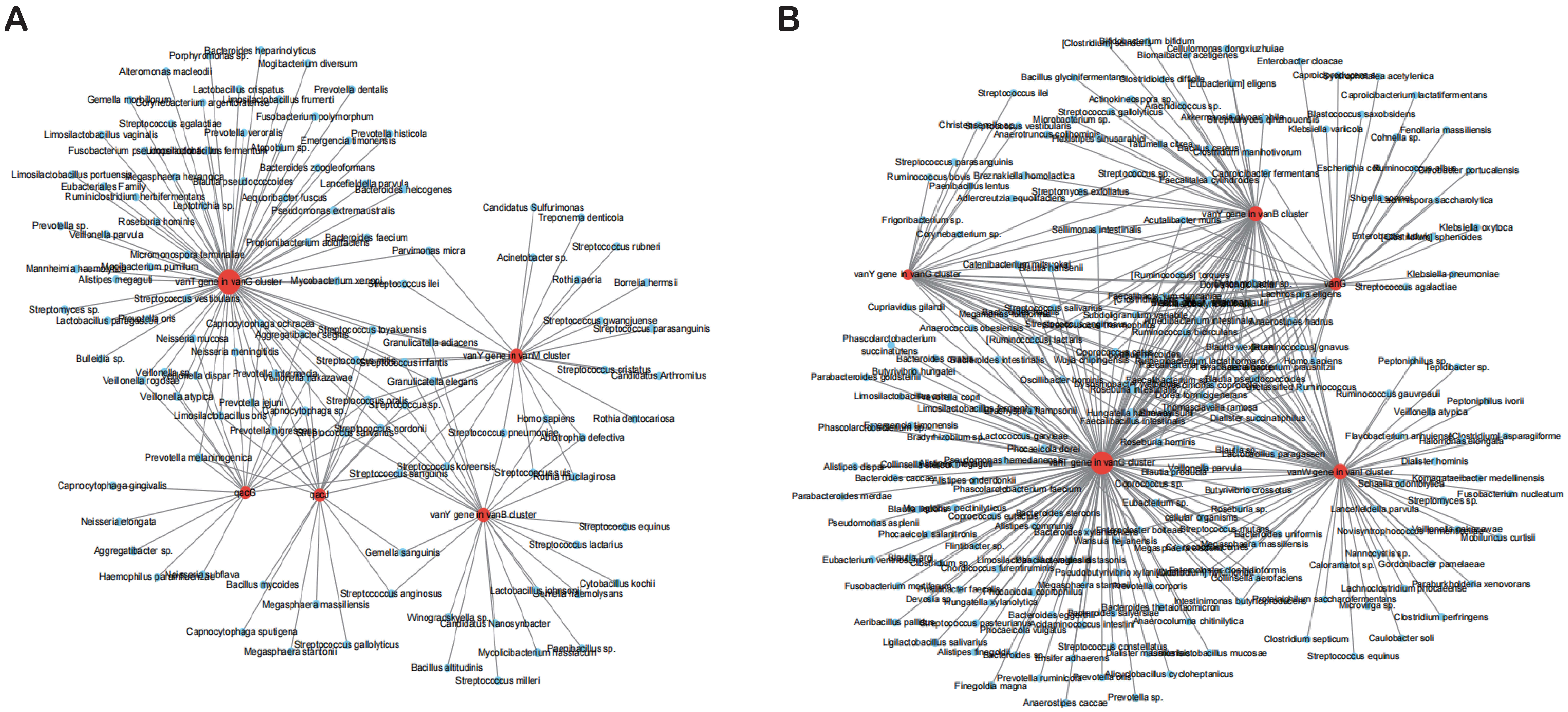
A
B
vanT gene in vanG cluster
vanY gene in vanM cluster
vanY gene in vanB cluster
qacB
qacJ
Candidatus Sulfurimonas
Treponema denticola
Streptococcus rubneri
Acinetobacter sp.
Rothia aeria
Borrelia hermsii
Streptococcus gwangjuense
Streptococcus parasanguinis
Granulicatella adiacens
Granulicatella elegans
Streptococcus cristatus
Candidatus Arthromitus
Homo sapiens
Rothia dentocariosa
Streptococcus pneumoniae
Abiotrophia defectiva
Streptococcus equinus
Streptococcus lactarius
Gemella sanguinis
Streptococcus anginosus
Cytobacillus kochii
Bacillus altitudinis
Streptococcus milleri
Capnocytophaga gingivalis
Neisseria elongata
Aggregatibacter sp.
Bacillus mycoides
Megasphaera massiliensis
Capnocytophaga sputigena
Megasphaera stantonii
Streptococcus gallolyticus
Prevotella melaninogenica
Bulleidia sp.
Veillonella atypica
Neisseria mucosa
Neisseria meningitidis
Capnocytophaga ochracea
Streptococcus vestibularis
Streptomyces sp.
Alistipes megaguti
Prevotella sp.
Veillonella parvula
Roseburia hominis
Leptotrichia sp.
Pseudomonas extremaustralis
Aequoribacter fuscus
Bacteroides zoogleoformans
Parvimonas micra
Mycobacterium xenopi
Streptococcus ilei
Prevotella dentalis
Prevotella histicola
Fusobacterium polymorphum
Mogibacterium diversum
Bacteroides heparinolyticus
Alteromonas macleodii
Lactobacillus crispatus
Streptococcus agalactiae
Limosilactobacillus vaginalis
Prevotella veroralis
Emergencia timonensis
Limosilactobacillus portuensis
Eubacteriales Family
Ruminiclostridium herbifermentans
vanY gene in vanB cluster
vanY gene in vanG cluster
vanG
vanY gene in vanI cluster
vanW gene in vanI cluster
Enterobacter cloacae
Bacillus glycinifermentans
Streptococcus ilei
Blastococcus saxobsidens
Klebsiella variicola
Fenollaria massiliensis
Cohnella sp.
Klebsiella oxytoca
Klebsiella pneumoniae
Streptococcus agalactiae
Peptoniphilus sp.
Tepidibacter sp.
Peptoniphilus ivorii
Veillonella atypica
Ruminococcus gauvreauii
Dialister hominis
Fusobacterium nucleatum
Streptomyces sp.
Lancefieldella parvula
Mobiluncus curtisii
Nannocystis sp.
Caloramator sp.
Gordonibacter pamelaeae
Paraburkholderia xenovorans
Microvirga sp.
Clostridium perfringens
Clostridium septicum
Caulobacter soli
Streptococcus equinus
Anaerostipes caccae
Prevotella sp.
Finegoldia magna
Ligilactobacillus salivarius
Aeribacillus pallidus
Fusobacterium mortiferum
Eubacterium ventriosum
Phocaeicola salanitronis
Pseudomonas asplenii
Parabacteroides merdae
Bacteroides caccae
Alistipes dispar
Phascolarctobacterium succinatutens
Parabacteroides goldsteinii
Cupriavidus gilardii
Frigoribacterium sp.
Corynebacterium sp.
Streptococcus parasanguinis
Ruminococcus bovis
Paenibacillus lentus
Adlercreutzia equolifaciens
Streptomyces exfoliatus
Sellimonas intestinalis
Acutalibacter muris
Roseburia hominis
Butyrivibrio crossotus
Coprococcus sp.
Eubacterium sp.
Roseburia sp.
Intestinimonas butyriciproducens
Anaerocolumna chitinilytica
Alicyclobacillus cycloheptanicus
Streptococcus constellatus
Shigella sonnei
Escherichia coli
